# Understanding real‐world treatment patterns and clinical outcomes in AL amyloidosis patients diagnosed in Canada: A population‐based cohort study

**DOI:** 10.1002/jha2.562

**Published:** 2022-09-05

**Authors:** Victor H. Jimenez‐Zepeda, Donna Reece, Rodrigo Rigo, Priyanka Gogna, Shiying Kong, Xun Yang Hu, Parv Chapani, Winson Y. Cheung, Darren R. Brenner, Richard Plante, Kun Shi, Asad Husain, Dipti Tankala, Devon J. Boyne

**Affiliations:** ^1^ Department of Medical Oncology and Hematology Tom Baker Cancer Centre Calgary Alberta Canada; ^2^ Division of Medical Oncology and Hematology Princess Margaret Cancer Centre Toronto Ontario Canada; ^3^ Oncology Outcomes Research Initiative University of Calgary Calgary Alberta Canada; ^4^ Department of Oncology, Cumming School of Medicine University of Calgary Calgary Alberta Canada; ^5^ Department of Public Health Sciences Queen's University Kingston Ontario Canada; ^6^ Department of Community Health Science University of Calgary Calgary Alberta Canada; ^7^ Department of Medicine, Cumming School of Medicine University of Calgary Calgary Alberta Canada; ^8^ Department of Medical Affairs, Janssen Toronto Ontario Canada; ^9^ Department of Market Access, Janssen Toronto Ontario Canada

**Keywords:** amyloidosis, epidemiology, health services research

## Abstract

Amyloid light chain (AL) amyloidosis is a rare and chronic bone marrow disorder. Existing claims data can be used to help understand the real‐world treatment patterns and outcomes of this patient population. Various population‐based administrative databases in Alberta, Canada were queried from 2010 to mid‐2019 to identify cases of AL amyloidosis. Baseline patient and disease characteristics, sequencing of pharmacologic therapies, overall survival, and healthcare resource utilization were evaluated. A total of 215 individuals with AL amyloidosis were included. Among patients diagnosed between 2012 and 2019, 149 (85.1%) initiated first‐line, 67 (38.3%) initiated second‐line, 22 (12.6%) initiated third‐line, and 11 (6.3%) initiated fourth‐line systemic therapy. In the first‐line setting, 99/149 (66.4%) received bortezomib, cyclophosphamide, and dexamethasone (CyBorD) and 21/149 (14.1%) received another bortezomib‐based regimen. Survival from time of diagnosis improved over time, with a median overall survival of 25.8 months (95% CI: 9.8, 57.1) for individuals diagnosed in 2010–2011 versus 52.1 months (95% CI: 25.6, NA) for those diagnosed in 2012–2019. Despite this improvement, the proportion of individuals diagnosed in 2012–2019 who survived beyond five‐years remained low (5‐year survival: 48.4%; 95% CI: 40.9, 57.2) which highlights an unmet need for more efficacious therapies.

## 1 INTRODUCTION

Amyloidosis results from the inability of certain proteins to retain a stable structure, which leads them to become amyloid fibrils in various tissues, including the heart, kidney, and liver [1, 2]. Over 25 different proteins have been described as amyloidogenic precursors [3]. Amyloid light chain (AL) amyloidosis is the most severe and common type of amyloidosis and is characterized by fibrils composed of a monoclonal immunoglobin light chain [4, 5]. AL amyloidosis typically manifests as a systemic disease, with local confinement of the disease to a single organ considered rare [1]. AL amyloidosis is related to multiple myeloma, another condition characterized by abnormal production of antibody‐producing cells [6]. There is substantial overlap between these two conditions, and patients are often diagnosed with both [6]. The main distinguishing factor between the two conditions is the type of cells involved; in AL amyloidosis, light chains are of primary concern, while in multiple myeloma, growth of abnormal cells in bone marrow are of primary concern [1, 2].

Both multiple myeloma and AL amyloidosis are commonly treated with chemotherapy alone, or in combination with stem‐cell transplantation [7]. Introduced in 2012, the primary chemotherapeutic regimen used to treat AL amyloidosis is bortezomib, cyclophosphamide, and dexamethasone (CyBorD) [8, 9, 10]. The introduction of this therapy has been attributed to improved survival and treatment outcomes in individuals with AL amyloidosis [1, 5]. Other pharmacologic therapies used to treat AL amyloidosis include cyclophosphamide, bortezomib, bendamustine, carfilzomib, lenalidomide, daratumumab, melphalan, pomalidomide, thalidomide, ixazomib, dexamethasone, prednisone, doxycycline, and methyldprednisolone [2].

Real‐world evidence pertaining to AL amyloidosis is limited, particularly in Canada. We previously described the characteristics and outcomes of 34 individuals diagnosed with AL Amyloidosis who were treated with CyBorD at a single center in Alberta, Canada [11, 12]. The purpose of this investigation was to build upon this prior work by leveraging population‐based administrative data to describe the characteristics, treatment patterns, and clinical outcomes of individuals diagnosed with AL amyloidosis in a Canadian real‐world setting.

## 2 METHODS

### 2.1 Study population

Population‐based administrative databases from Alberta, Canada were queried to identify individuals who were diagnosed with AL amyloidosis in the province between January 1, 2010 and June 30, 2019 using a modified version of the algorithm implemented in Quock et al. [13, 14]. Diagnostic fields within the Discharge Abstract Database, National Ambulatory Care Reporting System, and Practitioner Claims databases were searched for International Classification of Diseases (ICDs) codes used in the claims‐based algorithm developed by Quock et al. [13, 14]. Databases were deterministically linked using the provincial unique lifetime identifier number. Individuals were flagged as having AL amyloidosis if they had a single amyloidosis ICD code in any diagnostic field and had received one of the following guideline recommended AL amyloidosis pharmacologic therapies on or after the date of the earliest amyloidosis ICD code: cyclophosphamide, bortezomib, bendamustine, daratumumab, lenalidomide, melphalan, ixazomib, pomalidomide, thalidomide, and doxycycline. In contrast to Quock et al. [13, 14], our algorithm included daratumumab and ixazomib but excluded the following treatments, which can be administered solely for non‐AL amyloidosis indications: dexamethasone, prednisone, and methylprednisone. Diagnosis with AL amyloidosis was confirmed via chart review by a trained medical doctor for all individuals flagged by the administrative algorithm.

Baseline characteristics were assessed using a combination of medical chart review and administrative data. Date of diagnosis was defined according to the date abstracted from the medical chart. In situations where the date of diagnosis was unavailable in the medical chart, we imputed the earliest date of the amyloidosis ICD code from the administrative databases. Organ involvement at the time of diagnosis was also abstracted from medical charts and defined as follows: heart involvement – AL amyloid involvement on echocardiogram, magnetic resonance imaging, or biopsy or an N‐terminal prohormone of brain natriuretic peptide (NTproBNP) ≥ 650 ng/L; kidney involvement – AL amyloid involvement on biopsy or albuminuria > 500 mg in 24 h or less; liver involvement – AL amyloid involvement on biopsy, alkaline phosphatase level greater than two times the upper limit of normal, or presence of hepatomegaly [15, 16, 17]. Charlson comorbidities within the 12 months prior to the date of diagnosis was assessed using Alberta administrative data using the algorithm developed and validated by Hude et al. [18]. Neighborhood‐level household income and educational attainment data were captured using data from the national census. Date of birth and biological sex were ascertained via the population registry. Eastern Cooperative Oncology Group (ECOG) score, CRAB (hypercalcemia, renal failure, anemia, or bone disease) symptoms, and SLiM CRAB (light chains > 100, one or more focal lesions, bone marrow plasma cell level > 60%, or CRAB) symptoms at the time of diagnosis, as well as reasons for discontinuation of first‐line therapy (if available) were also extracted from the medical notes [19]. Concurrent multiple myeloma was defined as a multiple myeloma diagnosis recorded in the medical notes or reported to the provincial cancer registry occurring within 12 months prior to the date of diagnosis with AL amyloidosis or anytime during the follow‐up period post‐AL diagnosis.

### 2.2 Treatment and outcomes

Lines of pharmacologic therapy were identified using the Pharmaceutical Information Network database, which contains records from community pharmacies within the province. This database was queried for the following AL amyloidosis pharmacologic therapies: cyclophosphamide, bortezomib, bendamustine, carfilzomib, lenalidomide, daratumumab, melphalan, pomalidomide, thalidomide, ixazomib, dexamethasone, prednisone, doxycycline, and methylprednisolone [14, 20]. We also examined hospitalization and ambulatory care records for evidence of stem cell transplant using the following procedure codes: 1WY19HHXXA, 1WY19HHXXI, WY19HHXXJ, 1LZ19HHU7A, 1LZ19HHU7J, 1LZ19HHU8A, 1LZ19HHU8J, and 1WY19HHXXM.

The initial pharmacologic regimen was classified according to all AL amyloidosis pharmacologic therapies received within 60 days of initiating the first agent. Subsequent lines of therapy were defined as receipt of any of the agents listed above that were not within the initial regimen (except for doxycycline) or if there was a gap of more than 90 days between successive dispensations. Since it is not used to treat AL amyloidosis and can be administered for other purposes, receipt of prednisone alone was not classified as a line of therapy in accordance with advice of medical experts. The end date of each line of pharmacologic treatment was defined as the earliest of the following three possible dates: (1) the date of the last cycle of the line of therapy plus 28 days; (2) the date of starting a subsequent line of therapy; or (3) the date of death or administrative censoring.

### 2.3 Statistical analysis

Baseline patient and disease characteristics, sequencing of pharmacologic therapies, overall survival (OS), and time to next treatment or death (TTNT) were evaluated. Patients were followed from the date of diagnosis until death, the last interaction with the healthcare system, or December 31, 2019, whichever occurred first. Descriptive statistics were used to summarize baseline characteristics and treatment patterns. Given the introduction of CyBorD in 2012, treatment pattern analyses were restricted to individuals diagnosed between 2012 and 2019 [8, 9, 10]. Median OS and TTNT along with 95% confidence intervals were estimated from diagnosis and from the initiation of each line of therapy using the Kaplan–Meier method. Survival from initiation of first‐line therapy was also stratified by select baseline characteristics.

### 2.4 Healthcare resource utilization

Healthcare resource utilization of individuals with AL amyloidosis was quantified and compared to that of the general population with respect to hospitalizations, ambulatory care encounters, and health practitioner claims. Individuals with AL amyloidosis were matched on age and sex to members of the general population in a 1:4 ratio. The total number of healthcare encounters per patient was operationalized as the sum of the number of hospitalizations, number of encounters with ambulatory care services, and number of encounters with health practitioners. In these analyses, multiple health practitioner claims occurring on the same day were considered to constitute a single encounter. Index time zero for individuals with AL amyloidosis was defined as the calendar time of diagnosis. Index time zero for members of the general population was defined as the first encounter with healthcare services within the year corresponding to the date of diagnosis for the matched AL amyloidosis patient. Members of the general population who had no healthcare encounters within the year of diagnosis were not matched. The mean difference and standardized mean difference (SMD) in number of healthcare encounters was estimated. To account for a lack of independence due to matching, 95% confidence intervals were estimated using cluster‐robust variance estimation. In addition to the comparison with the general population, we also compared the mean number of healthcare encounters among AL amyloidosis who had concurrent multiple myeloma with those who did not have concurrent multiple myeloma. In these analyses, healthcare resource utilization was examined from the time of initial diagnosis with AL amyloidosis until the end of follow‐up.

### 2.5 Ethics statement

The Health Research Ethics Board of Alberta Cancer Committee approved this study (HREBA.CC‐20‐0481).

## 3 RESULTS

### 3.1 Baseline characteristics

A total of 6,238,529 unique individuals were queried, of which 622 were initially flagged as having a potential AL amyloidosis diagnosis. After chart review, 215 individuals were confirmed to have AL amyloidosis. The mean age at diagnosis was 66 years, the majority of patients were men (59.5%), and the mean neighborhood‐level household annual income was $43,698 (Table 1). At initial diagnosis, 35.8% of individuals had an ECOG performance status of 2 or greater, 84.7% had at least one comorbidity, and 29.8% had a concurrent multiple myeloma diagnosis. Kidney (55.8%) and cardiac (67.9%) involvement were more common at baseline than liver involvement (15.3%). Presence of one or more CRAB symptoms at diagnosis (hypercalcemia, renal failure, anemia, bone lesions) was reported in 40.9%.

**TABLE 1:** Baseline characteristics of AL amyloidosis patients diagnosed in Alberta, Canada between 2010 and mid‐2019 (*n* = 215)

Variable	Estimate (*n* = 215)
Age at diagnosis (mean [SD])	66.12 (11.52)
Age at diagnosis > 65 years (%)	116 (54.0)
Males (%)	128 (59.5)
Neighbourhood‐level household annual income, CAD (mean [SD])	$43,698 ($27,847)
Proportion of individuals in neighborhood who achieved a high‐school level education or greater (mean [SD])	0.78 (0.10)
ECOG performance status (%)	
0	40 (18.6)
1	91 (42.3)
2	53 (24.7)
3+	24 (11.2)
Missing	7 (3.3)
Number of comorbidities (%)	
0	33 (15.3)
1	61 (28.4)
2	61 (28.4)
3	31 (14.4)
4+	29 (13.5)
Concurrent multiple myeloma diagnosis (%)	64 (29.8)
Number of involved organs^a^ (%)	
0	28 (13.0)
1	89 (41.4)
2	75 (34.9)
3	18 (8.4)
Uncertain/Missing	5 (2.3)
Number of involved organs^2^ (median [IQR])	1.0 (1.0–2.0)
Cardiac involvement at baseline (%)	
No	91 (42.3)
Yes	120 (55.8)
Uncertain/missing	4 (1.9)
Kidney involvement at baseline (%)	
No	69 (32.1)
Yes	146 (67.9)
Liver involvement at baseline (%)	
No	180 (83.7)
Yes	33 (15.3)
Uncertain/missing	2 (0.9)
Bone marrow plasma cell level % (median [IQR])	10.0 (5.0–15.0)
Presence of CRAB symptoms (%)	
No	123 (57.2)
Yes	88 (40.9)
Missing	4 (1.9)

Abbreviations: CAD, Canadian Dollars; CRAB, calcium, liver failure, anemia, bone lesions; ECOG, Eastern Cooperative Oncology Group; IQR, interquartile range; SD, standard deviation.

^a^ Cardiac, kidney, and liver involvement.

### 3.2 Treatment patterns

Among individuals diagnosed between 2012 and 2019 (*n* = 175), 149 (85.1%) initiated first‐line pharmacologic therapy, 67 (38.3%) initiated second‐line therapy, 22 (12.6%) initiated third‐line therapy, and 11 (6.3%) initiated fourth‐line therapy (Table 2). In patients who initiated first‐line therapy (*n* = 149), 99 (66.4%) received CyBorD, and 21 (14.1%) received another Bortezomib‐based regimen. Other treatments received included melphalan and dexamethasone (MDex), lenalidomide and dexamethasone (RevDex), and dexamethasone (Dex) alone (*n* < 10). In patients who initiated second‐line therapy (*n* = 67), 20 (29.9%) received RevDex, 13 (19.4%) received another Bortezomib‐based regimen, 10 (14.9%) received CyBorD, 12 (17.9%) received Dex alone, and 12 (17.9%) received other treatments, including MDex. A total of 19 of the 215 (8.8%) individuals with AL amyloidosis received a stem cell transplant, and less than 10 received an organ transplant. A total of 33 of 215 (18.1%) patients received doxycycline currently with front‐line therapy.

**TABLE 2:** Types of systemic therapies used to treat individuals with AL amyloidosis who were diagnosed in Alberta, Canada between 2012 and 2019 (*n* = 175), stratified by line of therapy^a^, ^b^

Variable	Estimate (*n* = 175)
Number of lines of therapy (Median [IQR])	1.0 (1.0–2.0)
1 L pharmacologic therapy (%)	149 (85.1)
CyBorD	99 (66.4)
Other Bortezomib‐based regimen	21 (14.1)
Other	29 (19.5)
2 L pharmacologic therapy (%)	67 (38.3)
RevDex3	20 (29.9)
Other Bortezomib‐based regimen	13 (19.4)
CyBorD	10 (14.9)
Dex alone	12 (17.9)
Other	12 (17.9)
3 L pharmacologic therapy (%)	22 (12.6)
Rev‐based regimen	10 (45.5)
Other	12 (54.5)
4 L pharmacologic therapy (%)	11 (6.3)

Abbreviations: 2 L, Second line; 3 L, Third line; 4 L, Fourth Line; CyBorD, bortezomib, cyclophosphamide, and dexamethasone; Dex, dexamethasone; IL, First line; IQR, interquartile range; MDex, melphalan and dexamethasone; RevDex, lenalidomide and dexamethasone.

^a^ The percentage of patients who initiated different types of regimens were estimated as a proportion of those who initiated the corresponding line of pharmacologic therapy.

^b^ Lenalidomide + dexamethasone + ixazomib and lenalidomide + dexamethasone + prednisone.

The median duration of first‐line therapy was 4.0 months (IQR: 2.5–6.2), second‐line therapy was 2.8 months (IQR: 0.9–6.5), and third‐line therapy was 4.4 months (IQR: 2.7–7.6). In the first‐line setting, 44.8% of patients completed their intended duration of therapy. The primary reasons for discontinuation of first‐line therapy were toxicity (13.1%), death (11.7%), and progression (9.7%). The median time from diagnosis to first‐line therapy initiation was 1.1 months (IQR: 0.5–2.2), from initiation of first‐line to initiation of second‐line therapy was 9.4 months (IQR: 4.6–22.6), and from initiation of second‐line to initiation of third‐line therapy was 11.3 months (IQR: 6.4–19.8).

### 3.3 Patient outcomes

**FIGURE 1:**
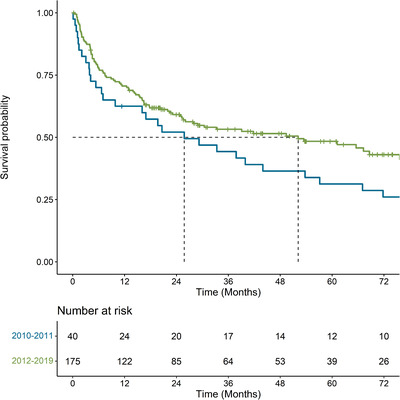
Kaplan–Meier curve for overall survival from time of diagnosis, stratified by period of initial diagnosis with AL amyloidosis among individuals diagnosed in Alberta, Canada between 2010 and mid‐2019 (*n* = 215)

**FIGURE 2:**
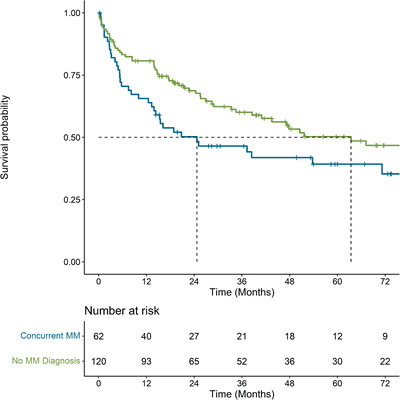
Kaplan–Meier curve for overall survival from the time of initiation of first line therapy, stratified by year of initial diagnosis with AL amyloidosis among individuals diagnosed in Alberta, Canada between 2010 and mid‐2019 and who initiated first‐line therapy (*n* = 182)

Median OS was 39.9 months (95% CI: 25.6, 67.0) from the time of diagnosis, 47.8 months (95% CI: 28.9, 79.5) from initiation of first‐line, 67.9 months (95% CI: 40.8, NA) from initiation of second‐line and 65.3 months (95% CI 62.6, NA) from initiation of third‐line. OS at 3, 6, 12, and 60 months from diagnosis and from initiation of each line of therapy are presented in Table 3. Median OS from initiation of first‐line was higher in patients 65+ compared to ≤65 years (79.5 months vs. 34.5 months), for females compared to males (79.5 months vs. 38.4 months), for patients diagnosed between 2012–2019 compared to 2010—2011 (52 months vs. 26 months), for patients with cardiac involvement compared to those without (102.8 months vs. 20.8 months), for patients with liver involvement compared to those without (53.8 months vs. 14.3 months), and for patients with a concurrent multiple myeloma diagnosis compared to those without (63.4 months vs. 24.7 months) (Table 4, Figures 1 and 2). Median OS was similar for people with and without liver involvement at diagnosis (53.8 months vs. 43.5 months; Table 4). TTNT results are available in the supplemental tables (Tables SI and SII).

**TABLE 3:** Overall survival from time of diagnosis and from initiation of each line of therapy among individuals with AL amyloidosis who were diagnosed in Alberta, Canada between 2010 and mid‐2019 (*n* = 215)

Time zero	Time point (Months)	Survival (95% CI)
Diagnosis	3	87.4 (83.0, 91.9)
	6	76.1 (70.6, 82.1)
	12	69.1 (63.1, 75.6)
	60	44.2 (37.5, 52.1)
Initiation L1	3	87.9 (83.2, 92.7)
	6	78.9 (73.2, 85.1)
	12	75.5 (69.5, 82.1)
	60	46.7 (39.2, 55.6)
Initiation L2	3	90.3 (84.1, 96.9)
	6	81.6 (73.7, 90.5)
	12	72.7 (63.5, 83.1)
	60	53.3 (42.6, 66.8)
Initiation L3	3	90.2 (80.3, 1.00)
	6	90.2 (80.3, 1.00)
	12	83.3 (70.9, 97.8)
	60	67.4 (51.7, 87.9)

Abbreviations: CI, confidence interval; L1, First line therapy; L2, Second line therapy; L3, Third line therapy.

**TABLE 4:** Overall survival from time of initiation of first‐line therapy among individuals with AL amyloidosis patients diagnosed in Alberta, Canada between 2010 and mid‐2019, stratified by baseline characteristics (*n* = 182)

Variable	Time point (months)	Survival (95% CI)
Age		
≤65 years	3	88.8 (82.5, 95.6)
	6	79.7 (71.8, 88.5)
	12	77.4 (69.2, 86.7)
	60	53.0 (42.8, 65.6)
65+ years	3	87.0 (80.3, 94.1)
	6	78.2 (70.1, 87.1)
	12	73.7 (65.2, 83.3)
	60	40.0 (29.9, 53.6)
Sex		
Female	3	94.3 (89, 99.9)
	6	82.7 (74.2, 92.1)
	12	82.7 (74.2, 92.1)
	60	51.1 (39.1, 66.7)
Male	3	83.8 (77.2, 90.9)
	6	76.5 (69.0, 84.8)
	12	71.0 (63.1, 80.0)
	60	44.0 (35.0, 55.4)
Period of diagnosis		
2010–2011	3	84.8 (73.5, 98.0)
	6	78.8 (66.0, 94.0)
	12	75.8 (62.5, 91.9)
	60	34.2 (21.0, 55.6)
2012–2019	3	88.5 (83.5, 93.8)
	6	78.9 (72.6, 85.8)
	12	75.5 (68.8, 82.8)
	60	50.8 (42.5, 60.7)
Kidney involvement		
No	3	80.0 (70.5, 90.8)
	6	66.2 (55.1, 79.4)
	12	64.4 (53.3, 77.9)
	60	42.0 (30.0, 58.8)
Yes	3	91.7 (87.0, 96.8)
	6	85.1 (79.0, 91.7)
	12	81.0 (74.3, 88.3)
	60	49.0 (40.0, 60.0)
Liver involvement		
No	3	89.4 (84.6, 94.5)
	6	82.0 (76.1, 88.4)
	12	78.0 (71.6, 84.9)
	60	48.9 (40.6, 58.9)
Yes	3	79.3 (65.9, 95.5)
	6	62.1 (46.7, 82.5)
	12	62.1 (46.7, 82.5)
	60	36.7 (22.5, 59.9)
Cardiac involvement		
No	3	92.3 (86.6, 98.4)
	6	89.7 (83.3, 96.7)
	12	85.9 (78.5, 94.0)
	60	61.6 (50.7, 74.9)
Yes	3	84.0 (77.1, 91.5)
	6	69.7 (61.2, 79.4)
	12	66.6 (57.9, 76.6)
	60	32.8 (23.7, 45.3)
MM diagnosis		
Concurrent MM	3	85.2 (76.8, 94.6)
	6	70.5 (59.9, 82.9)
	12	65.6 (54.7, 78.6)
	60	39.2 (28.0, 54.9)
No MM diagnosis	3	89.2 (83.8, 94.9)
	6	83.2 (76.8, 90.2)
	12	80.7 (73.9, 88.1)
	60	50.3 (41.0, 61.7)

Abbreviations: CI, confidence interval; MM, multiple myeloma.

### 3.4 Healthcare resource utilization

The average number of healthcare encounters within the observed follow‐up period for AL amyloidosis patients was significantly higher than that of age‐sex matched members of the general population (mean difference: 118.7 encounters [95% CI: 93.0, 144.3], SMD: 0.67 [95% CI: 0.52–0.81]) (Table 5). Individuals with concurrent multiple myeloma had comparable healthcare resource utilization to those who did not have concurrent multiple myeloma (mean difference: −49.8 encounters [95% CI: −135.3, 35.7], SMD: −0.18, [95% CI:−0.49–0.13] (Table 5). Healthcare resource utilization within different lines of therapy is presented in Table SIII.

**TABLE 5:** Comparison of healthcare resource utilization (mean events per patient) among individuals diagnosed with AL amyloidosis in Alberta, Canada between 2010 and mid‐2019 with age‐sex matched members of the general population and between individuals with and without concurrent multiple myeloma

	Mean number of encounters (SD)	Mean difference	Standardized mean difference
AL amyloidosis versus age‐sex matched general population			
AL Amyloidosis	199.4 (300.6)	118.7 (93.0, 144.3)^a^	0.7 (0.5, 0.8)^a^
General population	80.8 (118.7)	Ref.	Ref.
AL amyloidosis only			
Concurrent MM	161.0 (207.6)	−49.8 (−135.3, 35.7)	−0.2 (−0.5, 0.1)
No concurrent MM	210.7 (305.9)	Ref.	Ref.

Abbreviations: CI, confidence interval; MM, multiple myeloma; SMD, standardized mean difference.

^a^ Cluster robust variance estimation is used to account for bias in the estimation of the standard error due to clustering within the data caused by matching. Ignoring this lack of independence tends to result in the underestimation of the standard error (i.e., the 95% CIs tend to be too narrow).

## 4 DISCUSSION

Herein, we describe the treatment patterns and outcomes of a large, real‐world, Canadian cohort of AL amyloidosis patients. Between 2012 and 2019, 85% of individuals initiated some form of pharmacologic therapy, which was most commonly CyBorD or another bortezomib‐based regimen. Considerable attrition between lines was observed. As highlighted in previous real‐world studies, survival of AL amyloidosis has improved overtime, which is likely attributable to improved response rates achieved by CyBorD [9, 11, 17, 21]. Despite this improvement, our study suggests that long‐term OS remains poor, with only one in two patients surviving beyond 5‐years in the modern treatment era. These findings emphasize the unmet need for additional, more efficacious therapeutic options in this patient population. In addition, the healthcare resource utilization of individuals with AL amyloidosis was almost twice that of age‐sex matched members of the general population, which suggests considerable disease burden. Despite having a much shorter lifespan, the healthcare resource utilization of individuals with AL amyloidosis who had concurrent multiple myeloma was comparable to that of those who did not. These findings suggest that the rate of healthcare resource utilization may be particularly high for those with concurrent multiple myeloma since they achieved a similar level of healthcare resource utilization despite living for approximately half as long as their counterparts who did not have concurrent multiple myeloma.

Previous real‐world studies have reported comparable results with respect to treatment patterns and patient outcomes. Several studies have noted bortezomib‐based regimens as the predominant first‐line therapy, with a small proportion receiving stem cell transplant (<10%) [22, 23, 24]. With respect to OS, a large European retrospective cohort study of 2031 individuals reported a median OS of 50.1 months in the post‐2010 period, which was comparable to the median OS of 52.1 months observed in the post‐2011 period within the current study [24]. With respect to healthcare resource utilization, other investigations have similarly observed a high burden of disease with AL amyloidosis [22, 23]. For example, one investigation found that in the first year following relapse, average resource utilization per patient per month was 0.14 emergency room visits, 0.16 inpatient admissions, and 8.2 days per stay for inpatient admissions [23].

There are several strengths of this investigation. To our knowledge, this investigation is the first multicenter real‐world study of AL amyloidosis conducted in Canada. Second, we relied on population‐based data that capture information on all individuals in the province, regardless of treatment center or referral patterns. This reliance on population‐based data minimizes the risk of selection bias and enhances the external validity of these results. Last, this investigation had sufficient follow‐up such that we were able to examine long‐term survival outcomes, which have not been widely studied.

Our study has some limitations of note. First, we relied on a claims‐based algorithm to identify cases, which may have misclassified some individuals with AL amyloidosis. Specifically, there may have been individuals with concurrent multiple myeloma where an AL amyloidosis diagnosis was never documented since such a diagnosis would not change clinical practice. Second, an administrative data algorithm was used to classify regimen groupings and lines of therapy, which may have led to misclassification. To minimize the risk of such bias, the algorithm used in this investigation was developed in collaboration with clinicians who treats AL amyloidosis in Canada. Third, our query of pharmacologic therapies was restricted to guideline recommended therapies that were administered in community pharmacies. This restriction may have missed therapies administered outside of the community pharmacy, including treatments that may have been administered in a patient support program or clinical trial. Last, comparisons of outcomes between those with a concurrent multiple myeloma diagnosis versus no diagnosis are prone to immortal‐time bias due to the definition of concurrent multiple myeloma. However, such bias would attenuate the association by giving an artificial survival advantage to the concurrent multiple myeloma cohort. Therefore, the estimates generated within our study are likely conservative in that they likely underestimate the true burden of concurrent multiple myeloma in this patient population.

In conclusion, this investigation provides population‐based real‐world evidence pertaining to the management and outcomes of AL amyloidosis patients in Canada. CyBorD and other bortezomib‐based regimens were found to be the primary frontline treatment modality used since 2012. High rates of attrition were observed between lines, with less than half of patients receiving two lines of therapy. Despite meaningful improvements in mortality over time, long‐term survival remained poor, and the healthcare resource utilization was high, which highlights an unmet need and a high disease burden in this patient population.

## AUTHOR CONTRIBUTIONS

Victor H. Jimenez‐Zepeda, Donna Reece, Darren R Brenner, Winson Y Cheung, Richard Plante, Kun Shi, Asad Husain, Eric Ammann, Dipti Tankala, and Devon J Boyne conceptualized the study design. Winson Y. Cheung, Rodrigo Rigo, Xun Yang Hu, Parv Chapani, and Shiying Kong performed the data collection and data preparation. Shiying Kong and Devon J. Boyne conducted the statistical analyses. Priyanka Gogna and Devon J Boyne wrote the manuscript. All co‐authors critically reviewed the manuscript and assisted with the interpretation of results

## CONFLICT OF INTEREST

Victor H. Jimenez‐Zepeda received honoraria from Janssen Inc. Donna Reece received honoraria and research funding from Janssen Inc. Rodrigo Rigo, Priyanka Gogna, Shiying Kong, Xun Yang Hu, Parv Chapani, Winson Y Cheung, Darren R. Brenner, and Devon J. Boyne are team members of Oncology Outcomes, which received research funding from Janssen Inc. Richard Plante, Kun Shi, Asad Husain, Eric Ammann, and Dipti Tankala are employees of Janssen Inc. Eric Amman owns shares in Janssen Inc.

## Supporting information

Supporting Information

## Data Availability

Data from this investigation are not available for sharing due to data privacy legislation.
