# Supplementary material for: Understanding real‐world treatment patterns and clinical outcomes in AL amyloidosis patients diagnosed in Canada: A population‐based cohort study
Source: EJHaem. 2022 Sep 5;3(4):1262–9. doi: 10.1002/jha2.562 (PMC9713227; doi:10.1002/jha2.562)
Supplement: Supplementary file 1 — Supporting Information [file JHA2-3-1262-s001.docx]

**Understanding real-world treatment patterns and clinical outcomes in AL amyloidosis patients in a population-based cohort – Supplemental File**

Table SI. Time to next treatment in AL amyloidosis patients based on initiation and lines of therapy

| **Time Zero** | **Time Point**  **(Months)** | **Survival (95% CI)** |
| --- | --- | --- |
| Initiation L1 | 3 | 84.0 (78.8, 89.5) |
|  | 6 | 67.8 (61.3, 75.0) |
|  | 12 | 50.9 (44.1, 58.8) |
|  | 60 | 15.4 (10.2, 23.2) |
| Initiation L2 | 3 | 86.6 (79.6, 94.3) |
|  | 6 | 74.2 (65.3, 84.4) |
|  | 12 | 55.0 (45.0, 67.1) |
|  | 60 | 26.4 (17.6, 39.8) |
| Initiation L3 | 3 | 90.2 (80.3, 1.00) |
|  | 6 | 80.2 (67.2, 95.7) |
|  | 12 | 59.4 (44.0, 80.1) |
|  | 60 | 20.7 (8.60, 49.8) |

Table SII. Time to next treatment in AL amyloidosis patients by baseline characteristics from initiation of first-line therapy

| **Variable** | **Median TTNT,**  **Months (95% CI)** |
| --- | --- |
| Age |  |
| < 65 years | 10.7 (7.4, 20.2) |
| 65+ years | 13.3 (10.0, 22.7) |
| Sex |  |
| Female | 14.0 (11.5, 27.8) |
| Male | 10.7 (7.8, 21.4) |
| Era |  |
| 2010-2011 | 7.4 (4.1, 22.8) |
| 2012-2019 | 13.3 (10.3, 22.6) |
| Kidney Involvement |  |
| No | 9.6 (5.4, 21.4) |
| Yes | 13.9 (10, 22.8) |
| Liver Involvement |  |
| No | 14.6 (10.7, 22.6) |
| Yes | 4.2 (3.1, 12.5) |
| Cardiac Involvement |  |
| No | 15.7 (10.0, 25.5) |
| Yes | 10.3 (6.4, 17.4) |
| MM Diagnosis |  |
| No | 6.6 (4.4, 12.5) |
| Yes | 18.6 (12.5, 23.6) |

L1 – First line therapy, L2 – Second line therapy, L3 – Third line therapy, CI – confidence interval

Table SIII. Healthcare resource utilization (mean events per patient) during each line of therapy among individuals diagnosed with AL amyloidosis in Alberta, Canada between 2010 and mid-2019

|  | **Health State** | | | |
| --- | --- | --- | --- | --- |
|  | **During 1L (n=182)** | **During 2L (n=85)** | **During 3L (n=31)** | **During 4L (n=15)** |
| Mean Events per Patient |  |  |  |  |
| Hospitalizations | 0.8 | 0.5 | 0.7 | <10 |
| Days hospitalized | 12.0 | 11.6 | 7.1 | 1.9 |
| Ambulatory Care  Encounters | 13.8 | 10.1 | 20.3 | 11.9 |
| Emergency | 1.5 | 1.1 | 1.5 | 1.3 |
| Non-emergency | 12.3 | 8.9 | 18.8 | 10.5 |
| Practitioner claims | 42.8 | 35.7 | 41.1 | 27.1 |
| Practitioner encounters | 24.6 | 22.9 | 27.1 | 17.7 |

Abbreviations: L1 – First line therapy, L2 – Second line therapy, L3 – Third line therapy
